# Supplementary material for: FANCM limits ALT activity by restricting telomeric replication stress induced by deregulated BLM and R-loops
Source: Nat Commun. 2019 May 28;10:2253. doi: 10.1038/s41467-019-10179-z (PMC6538666; doi:10.1038/s41467-019-10179-z)
Supplement: Supplementary file 1 — Supplementary Information [file 41467_2019_10179_MOESM1_ESM.pdf]

SUPPLEMENTARY INFORMATION FOR

FANCM limits ALT activity by restricting telomeric replication stress induced by deregulated BLM and R-loops

Silva et al.

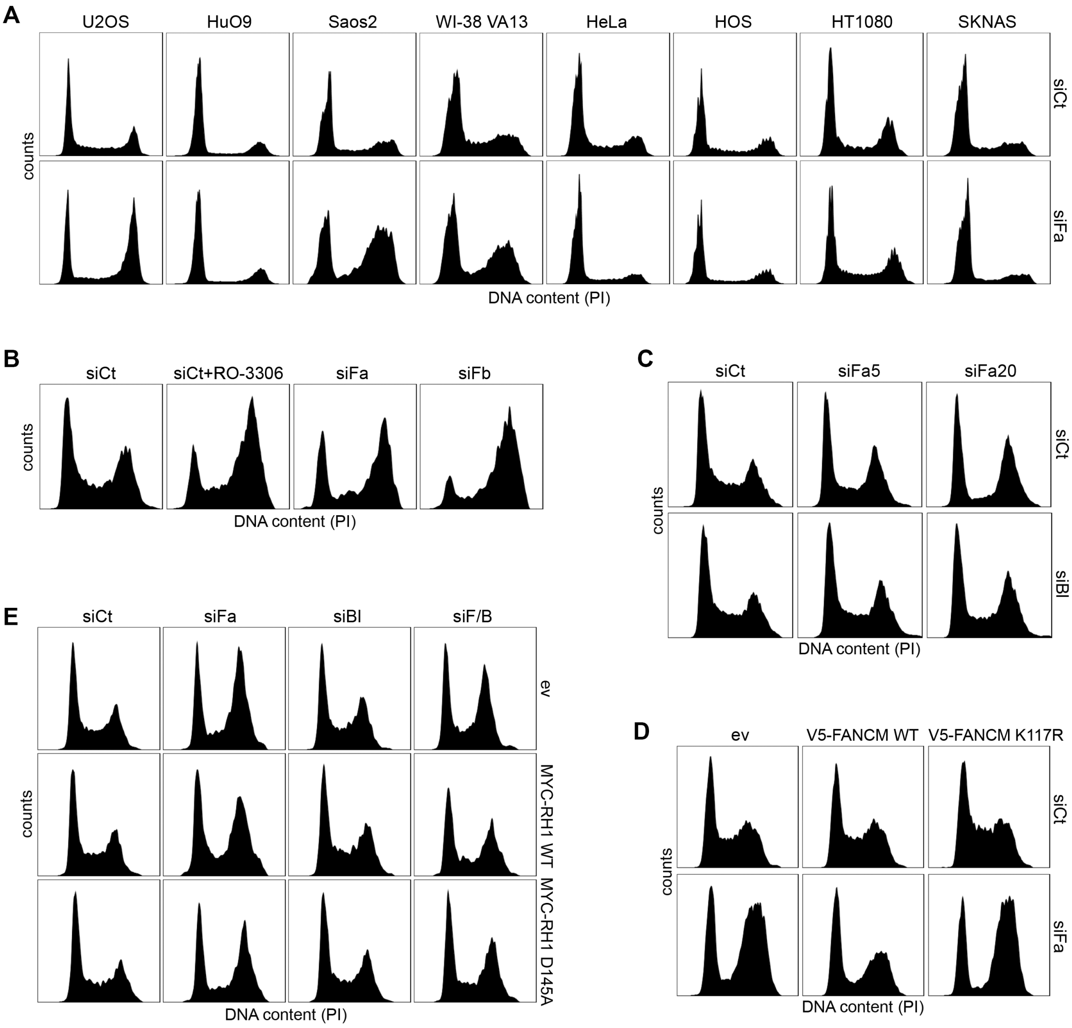

Supplementary Figure 1. Representative examples of cell cycle profiles of cells used in this study. (**A-E**) Cells were fixed and permeabilized with ethanol, stained with propidium iodide (PI) and FACS analyzed. Cell counts (y axis) are plotted against PI intensity (x axis).

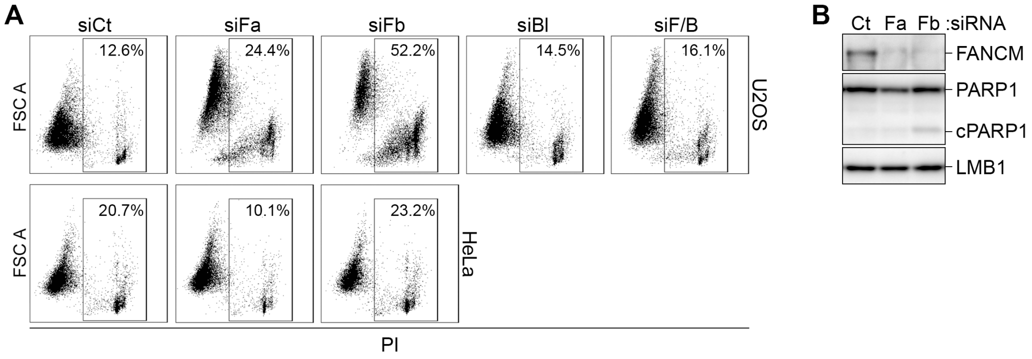

Supplementary 2. FANCM depletion induces cell death in U2OS cells. (**A**) U2OS and HeLa cells were transfected with the indicated siRNAs and harvested for PI staining without permeabilization 3 days after transfection. Forward Scatter A (FSCA; y axis) is plotted against PI intensity (x axis). Numbers are fractions of cells positive to PI staining as defined by the indicated gate. (**B**) Western blot analysis of U2OS cells treated as in **A**. cPARP1: cleaved PARP1. Lamin B1 (LMB1) serves as a loading control.

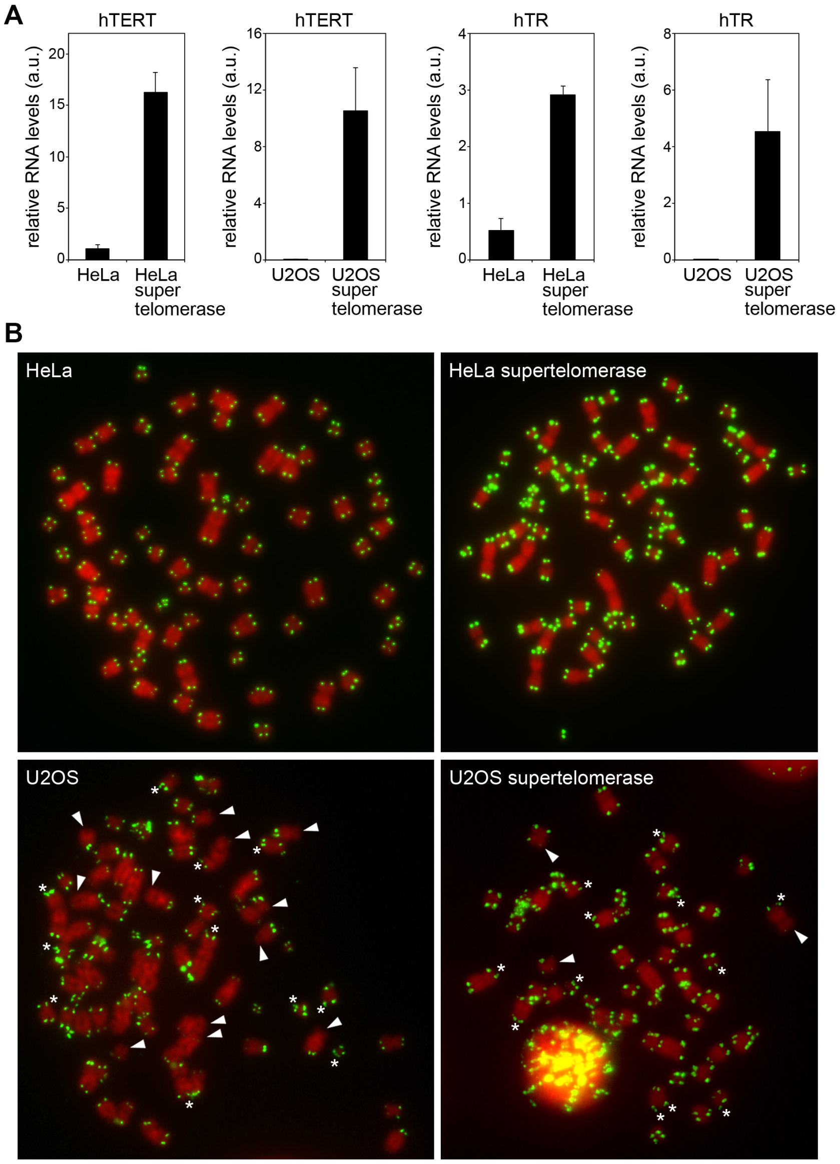

Supplementary Figure 3. Validation of telomerase activity in supertelomerase cells. HeLa and U2OS cells were stably infected with retroviruses expressing the catalytic (hTERT) and RNA (hTR) subunits of telomerase. Stable populations grown in culture for at least 2 months were used for validation experiments. (**A**) Quantitative RT-PCR of hTERT and hTR using total RNA. Values for supertelomerase cells are relative to uninfected cells. Bars and error bars are means and SDs from 3 independent experiments. (**B**) Examples of telomeric FISH experiments on metaphases from the indicated cell lines. Telomeric repeats are in green, DAPI-stained DNA in red. Note the strongly increased telomeric signal in HeLa supertelomerase cells, and the diminished incidence of telomere free ends (white arrowheads) but not of fragile telomeres (white asterisks) in U2OS supertelomerase cells. Source data are provided as a Source Data file.

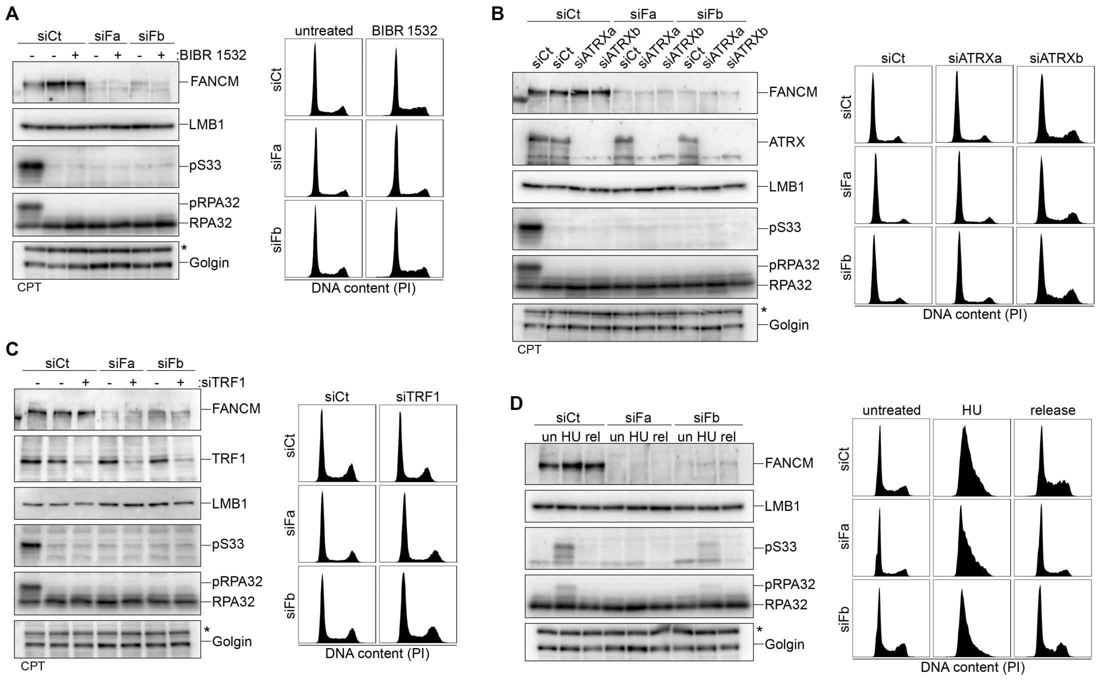

Supplementary Figure 4. Telomerase inhibition, ATRX depletion and telomeric or generalized replication stress do not sensitize HeLa cells to FANCM depletion. HeLa cells were treated as indicated and subjected to western blot (left of each panel) and PI/FACS analysis (right). pS33: RPA32 phosphorylated at serine 33, pRPA32: phosphorylated RPA32. LMB1 and Golgin serve as loading controls. As a control for pS33 activation, cells were treated with camptothecin (CPT). The asterisks indicate a band cross-reacting with the anti-Golgin antibody. (**A**) Cells were treated with BIBR 1532 for 7 days and with siRNAs for 3 days. (**B**) Cells were transfected with two independent siRNAs against ATRX and 3 days later transfected again with the same siRNAs and with FANCM siRNAs. (**C**) Cells were treated with siRNAs for 3 days. (**D**) Cells were treated with siRNAs for 3 days, blocked with hydroxyurea (HU) for 16 hours and release (rel) into the cell cycle for 18 hours by washing HU off the culture medium.

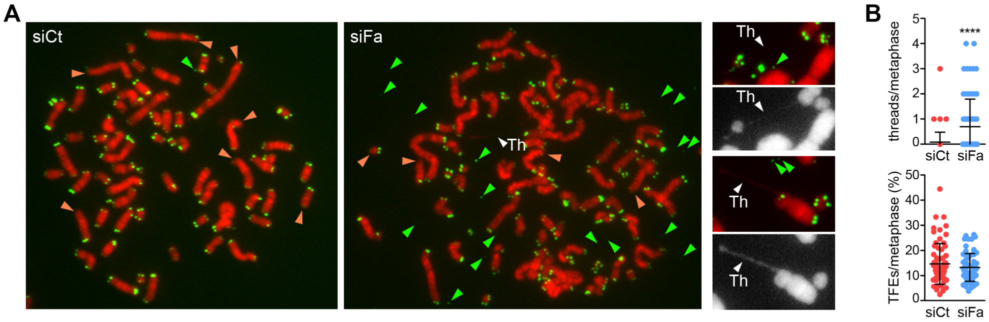

Supplementary Figure 5. FANCM suppresses ECTRs and DNA threads in U2OS cells. (**A**) Examples of telomeric DNA FISH on metaphases from U2OS cells transfected with the indicated siRNAs and harvested 48 hours after transfection. Telomeric repeats are in green, DAPI-stained DNA in red or grey. Green arrowheads point to ECTRs, white arrowheads to DNA threads (Th) and red arrowheads to telomere free ends (TFEs). Enlarged examples are shown on the right, with DAPI stained DNA also shown in grey on a black background to facilitate visualization of DNA threads. (**B**) Quantifications of numbers of DNA threads per metaphase or fraction (%) of TFEs per metaphase in cells as in A. A total of 65-75 metaphases from three independent experiments were analyzed for each sample. *P* values were calculated with a Mann-Whitney *U* test. ****$P < 0.0001$. Source data are provided as a Source Data file.

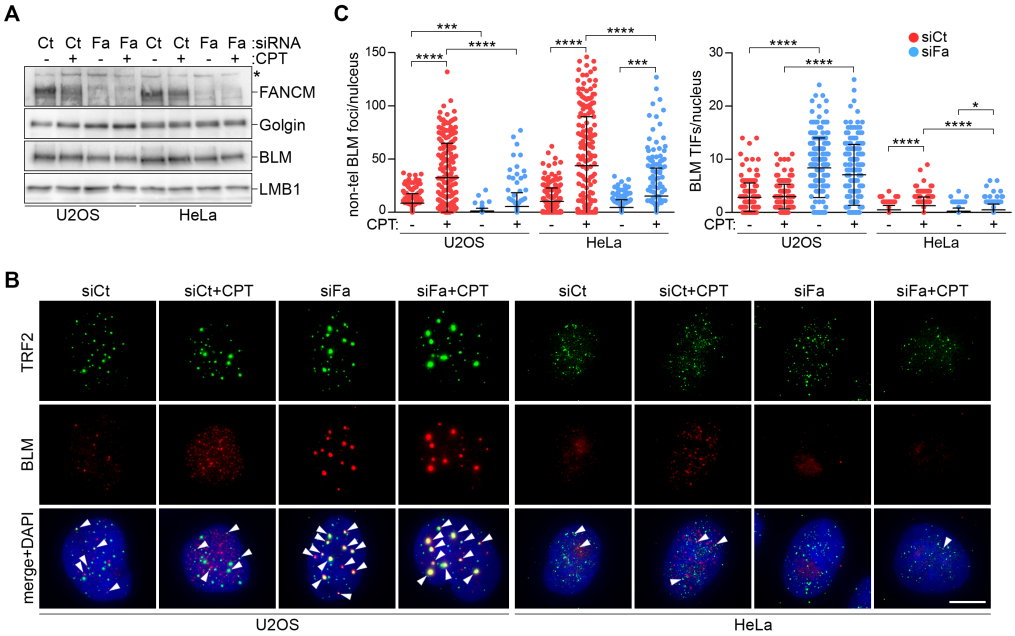

Supplementary Figure 6. FANCM depletion leads to BLM recruitment to telomeres in ALT cells but prevents BLM recruitment to CPT-induced DNA damage sites. (**A**) Western blot analysis of FANCM and BLM protein levels in U2OS and HeLa cells transfected and treated with camptothecin (CPT) as indicated. Cells were harvested 48 hours after transfection. Golgin and LMB1 serve as loading controls. (**B**) Examples of BLM immunostaining (red) combined with TRF2 immunostaining (green) on cells as in **A**. In the merge panel, DAPI-stained DNA is also shown (blue). Arrowheads point to BLM TIFs. Scale bar: 10 μm. (**C**) Quantifications of numbers of BLM foci accumulating outside telomeres (non-tel) and of BLM TIFs per nucleus in experiments as in **B**. A total of at least 179 nuclei from two independent experiments were analyzed for each sample. Bars and error bars are means and SDs. *P* values were calculated with a two-way ANOVA followed by Tukey's HSD. **P* < 0.05, ****P* < 0.001, *****P* < 0.0001. Source data are provided as a Source Data file.

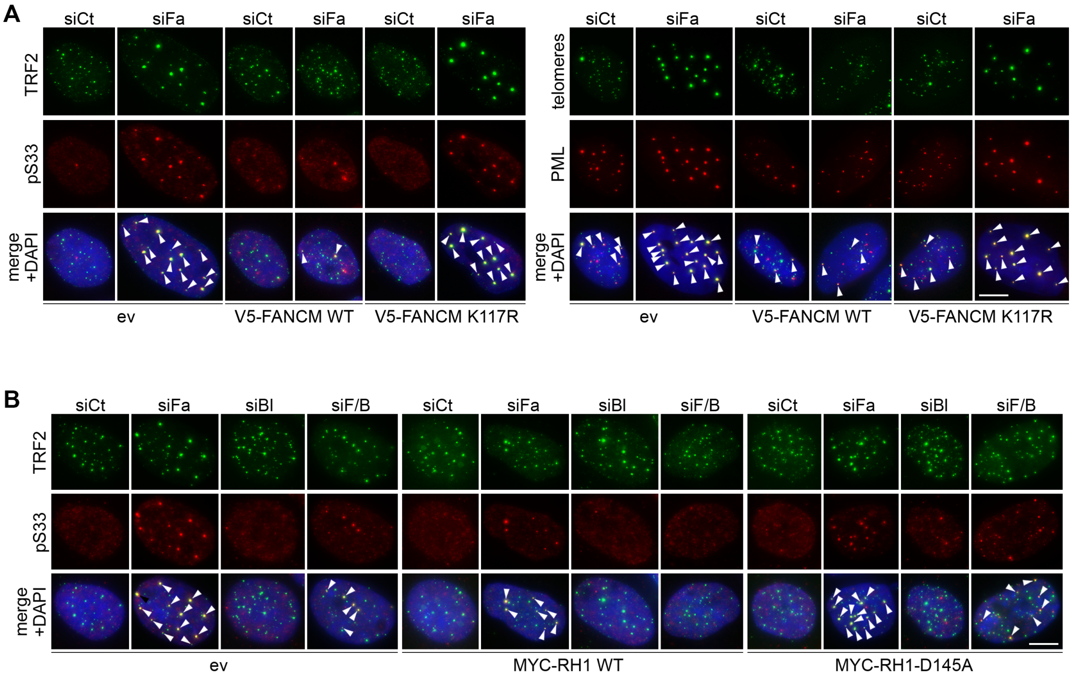

Supplementary Figure 7. The replication stress induced by FANCM depletion is averted by FANCM ATPase/translocase activity, depletion of BLM and overexpression of RNaseH1. (**A**) Examples of pS33 immunostaining (red) combined with TRF2 immunostaining (green) and of PML immunostaining (red) combined with telomeric FISH (green) in U2OS cells infected with retroviruses expressing V5 epitope-tagged FANCM variants or empty vector (ev) control retroviruses. WT: wild type, K117R: ATPase/translocase dead FANCM. (**B**) Examples of pS33 immunostaining (red) combined with TRF2 immunostaining (green) in U2OS cells infected with retroviruses expressing MYC epitope-tagged RNaseH1 (RH1) variants or ev control retroviruses. D145A: endoribonuclease dead RNaseH1. In all cases, five days after infections cells were transfected with the indicated siRNAs and harvested 48 hours later. siF/B: combined siFa and siBl. In the merge panel, DAPI-stained DNA is also shown (blue). Arrowheads point to colocalization events. Scale bar: 10 μm.
