# Supplementary material for: FANCM limits ALT activity by restricting telomeric replication stress induced by deregulated BLM and R-loops
Source: Nat Commun. 2019 May 28;10:2253. doi: 10.1038/s41467-019-10179-z (PMC6538666; doi:10.1038/s41467-019-10179-z)
Supplement: Supplementary file 3 — Reporting Summary [file 41467_2019_10179_MOESM3_ESM.pdf]

nature research

Corresponding author(s): Claus M. Azzalin

Reporting Summary

Nature Research wishes to improve the reproducibility of the work that we publish. This form provides structure for consistency and transparency in reporting. For further information on Nature Research policies, see Authors & Referees and the Editorial Policy Checklist.

Statistical parameters

When statistical analyses are reported, confirm that the following items are present in the relevant location (e.g. figure legend, table legend, main text, or Methods section).

n/a	Confirmed	
☐	☒	The exact sample size (*n*) for each experimental group/condition, given as a discrete number and unit of measurement
☐	☒	An indication of whether measurements were taken from distinct samples or whether the same sample was measured repeatedly
☐	☒	The statistical test(s) used AND whether they are one- or two-sided<br>*Only common tests should be described solely by name; describe more complex techniques in the Methods section.*
☒	☐	A description of all covariates tested
☒	☐	A description of any assumptions or corrections, such as tests of normality and adjustment for multiple comparisons
☐	☒	A full description of the statistics including central tendency (e.g. means) or other basic estimates (e.g. regression coefficient) AND variation (e.g. standard deviation) or associated estimates of uncertainty (e.g. confidence intervals)
☒	☐	For null hypothesis testing, the test statistic (e.g. *F*, *t*, *r*) with confidence intervals, effect sizes, degrees of freedom and *P* value noted<br>*Give P values as exact values whenever suitable.*
☒	☐	For Bayesian analysis, information on the choice of priors and Markov chain Monte Carlo settings
☒	☐	For hierarchical and complex designs, identification of the appropriate level for tests and full reporting of outcomes
☒	☐	Estimates of effect sizes (e.g. Cohen's *d*, Pearson's *r*), indicating how they were calculated
☐	☒	Clearly defined error bars<br>*State explicitly what error bars represent (e.g. SD, SE, CI)*

Our web collection on statistics for biologists may be useful.

Software and code

Policy information about availability of computer code

Data collection	Olympus IX 81 or Zeiss Cell Observer e for microscopy; Typhoon FLA 7000 Control Software and AlphaView (FluorChem HD2) for acquirement of radioactive and chemiluminescent signals, respectively.
Data analysis	ImageJ and Adobe Photoshop for image processing; R, Microsoft Excel and Graph Pad Prism for graph plotting and statistical analysis.

For manuscripts utilizing custom algorithms or software that are central to the research but not yet described in published literature, software must be made available to editors/reviewers upon request. We strongly encourage code deposition in a community repository (e.g. GitHub). See the Nature Research guidelines for submitting code & software for further information.

Data

Policy information about availability of data

All manuscripts must include a data availability statement. This statement should provide the following information, where applicable:

- Accession codes, unique identifiers, or web links for publicly available datasets
- A list of figures that have associated raw data
- A description of any restrictions on data availability

Original/raw data are presented in Source data files. Additional data are available from the corresponding author on request. Also

Field-specific reporting

Please select the best fit for your research. If you are not sure, read the appropriate sections before making your selection.

☒ Life sciences ☐ Behavioural & social sciences ☐ Ecological, evolutionary & environmental sciences

For a reference copy of the document with all sections, see nature.com/authors/policies/ReportingSummary-flat.pdf

Life sciences study design

All studies must disclose on these points even when the disclosure is negative.

Sample size	Sample sizes were chosen according to commonly used and accepted standards in the field. No statistical method was used to pre-determine sizes. The numbers of independent experiments (n) are indicated in the Methods section and in figure legends.
Data exclusions	No data were excluded from any analysis, except for EdU detection experiments, where only cell with a maximum of 25 punctate EdU signals were considered.
Replication	All experiments were reliably reproduced. At least three independent biological replicates were performed unless clearly stated in the figure legend.
Randomization	No randomization was used.
Blinding	Blinding was not used for data analysis.

Reporting for specific materials, systems and methods

Materials & experimental systems

n/a	Involved in the study
☒	☐ Unique biological materials
☐	☒ Antibodies
☐	☒ Eukaryotic cell lines
☒	☐ Palaeontology
☒	☐ Animals and other organisms
☒	☐ Human research participants

Methods

n/a	Involved in the study
☒	☐ ChIP-seq
☐	☒ Flow cytometry
☒	☐ MRI-based neuroimaging

Antibodies

Antibodies used	Antibodies used for western blotting: mouse monoclonal anti-FANCM (CV5.1, 1:1000 dilution); mouse monoclonal anti-Golgin 97 (Molecular Probes, A-21270, 1:5000 dilution); rabbit polyclonal anti-KAP1 (Bethyl Laboratories, A300-274A, 1:2000 dilution); rabbit polyclonal anti-pKAP1 (Ser 824) (Bethyl Laboratories, A300-767A, 1:2000 dilution); mouse monoclonal anti-CHK1 (Santa Cruz Biotechnology, sc-8408, 1:1000 dilution); rabbit monoclonal anti-pCHK1 Ser 345 (Cell Signaling, 2348, 1:500 dilution); rabbit polyclonal anti-RPA32 (Bethyl Laboratories, A300-244A, 1:3000 dilution); rabbit polyclonal anti-pRPA32 Ser 33 (Bethyl Laboratories, A300-246A, 1:1000 dilution); rabbit polyclonal anti-Lamin B1 (GeneTex, GTX103292, 1:1000 dilution); rabbit polyclonal anti-RNaseH1 (GeneTex, GTX117624, 1:500 dilution); mouse monoclonal anti-beta Actin (Abcam, ab8224, 1:5000 dilution); rabbit polyclonal anti-BLM (Bethyl Laboratories, A300-110A, 1:3000 dilution); rabbit polyclonal anti-PML (a kind gift from M. Carmo-Fonseca, iMM, Lisbon, Portugal, 1:1000 dilution); rabbit polyclonal anti-PARP1 (Cell Signaling, 9542, 1:1000 dilution); mouse monoclonal anti-POLD3 (Novus Biologicals, H00010714-M01, 1:500 dilution); rabbit polyclonal anti-ATRX (Bethyl Laboratories, A301-045A-T, 1:1000 dilution); sheep polyclonal anti-TRF1 (R&D Systems, AF5300, 1:1000 dilution). Secondary antibodies were HRP-conjugated goat anti-mouse and goat anti-rabbit IgGs (Bethyl Laboratories, A90-116P and A120-101P, 1:2000 dilution) and HRP-conjugated donkey anti-sheep IgG (Novus Bio, NBP1-75437, 1:3000 dilution).<br>Antibodies used for indirect immunofluorescence: rabbit polyclonal anti-pRPA32 pSer 33 (Bethyl Laboratories, A300-246A, 1:1000 dilution); rabbit polyclonal anti-53BP1 (Abcam, ab21083, 1:1000 dilution); mouse monoclonal anti-TRF2 (Millipore, 05-521, 1:500 dilution); rabbit polyclonal anti-BLM (Bethyl, A300-110A, 1:5000 dilution); rabbit polyclonal anti-PML (a kind gift from M. Carmo-Fonseca, iMM, Lisbon, Portugal, 1:500 dilution); mouse monoclonal anti-RAD51 (Abcam, ab213, 1:100 dilution); mouse monoclonal anti-POLD3 (Novus Biologicals, H00010714-M01, 1:100 dilution); rabbit polyclonal anti-RAP1 (Bethyl, A300-306A, 1:500 dilution). Secondary antibodies were Alexa Fluor 568-conjugated donkey anti-rabbit IgGs (Thermo Fisher Scientific, A10042) and Alexa Fluor 488-conjugated donkey anti-mouse IgGs (Thermo Fisher Scientific, A21202).<br>Antibodies used for ChIP and DRIP:mouse monoclonal anti FANCM (CE56.1) and S9.6, respectively.

Validation	The specificity of FANCM antibodies was confirmed by western blotting using extracts from siRNA depleted cells. All other antibodies were commercial and validated in previous published papers or by the company.

Eukaryotic cell lines

Policy information about cell lines

Cell line source(s)	HeLa cervical cancer, HT1080 fibrosarcoma, and HEK293 embryonic kidney cells were purchased from ATCC. U2OS osteosarcoma cells were a kind gift from M. Lopes (IMCR, Zurich, Switzerland). HuO9, Saos2 and HOS osteosarcoma cells were a kind gift from B. Fuchs (Balgrist University Hospital, Zurich, Switzerland). WI-38 VA13 in vitro SV40-transformed lung fibroblasts were a kind gift from A. Londoño-Vallejo (CNRS, Paris, France). SKNAS neuroblastoma cells were a kind gift from O. Shakhova (University Hospital Zurich, Switzerland).
Authentication	Telomere length and ALT-associated promyelocytic leukaemia body (APB) analyses confirmed wether cells were ALT or telomerase positive.
Mycoplasma contamination	All cells were mycoplasma negative. Mycoplasma contaminations were tested using the VenorGeM Mycoplasma PCR Detection Kit (Minerva Biolabs) according to the manufacturer's instructions.
Commonly misidentified lines (See ICLAC register)	None of the cell lines is listed as commonly misidentified at ICLAC.

Flow Cytometry

Plots

Confirm that:

☒ The axis labels state the marker and fluorochrome used (e.g. CD4-FITC).

☒ The axis scales are clearly visible. Include numbers along axes only for bottom left plot of group (a 'group' is an analysis of identical markers).

☒ All plots are contour plots with outliers or pseudocolor plots.

☒ A numerical value for number of cells or percentage (with statistics) is provided.

Methodology

Sample preparation	Cells were trypsinized and pelleted by centrifugation at 500 g at 4°C for 5 min. Cell pellets were fixed in 70% ethanol at -20°C for 30 min, treated with 25 µg/ml RNaseA (Sigma-Aldrich) in 1x PBS at 37°C for 20 min, and stained with 20 µg/ml propidium iodide (Sigma-Aldrich) in 1x PBS at 4°C for 10 min. For cell viability assays, cells were stained with propidium iodide in a bsece of previous permeabilization with ethanol.
Instrument	BD FACSCalibur or BD Accuri C6 (BD Biosciences).
Software	FlowJo
Cell population abundance	n/a
Gating strategy	n/a

☐ Tick this box to confirm that a figure exemplifying the gating strategy is provided in the Supplementary Information.
