# Supplementary material for: FANCM limits ALT activity by restricting telomeric replication stress induced by deregulated BLM and R-loops
Source: Nat Commun. 2019 May 28;10:2253. doi: 10.1038/s41467-019-10179-z (PMC6538666; doi:10.1038/s41467-019-10179-z)

Uncropped blots (related to Fig. 1a)

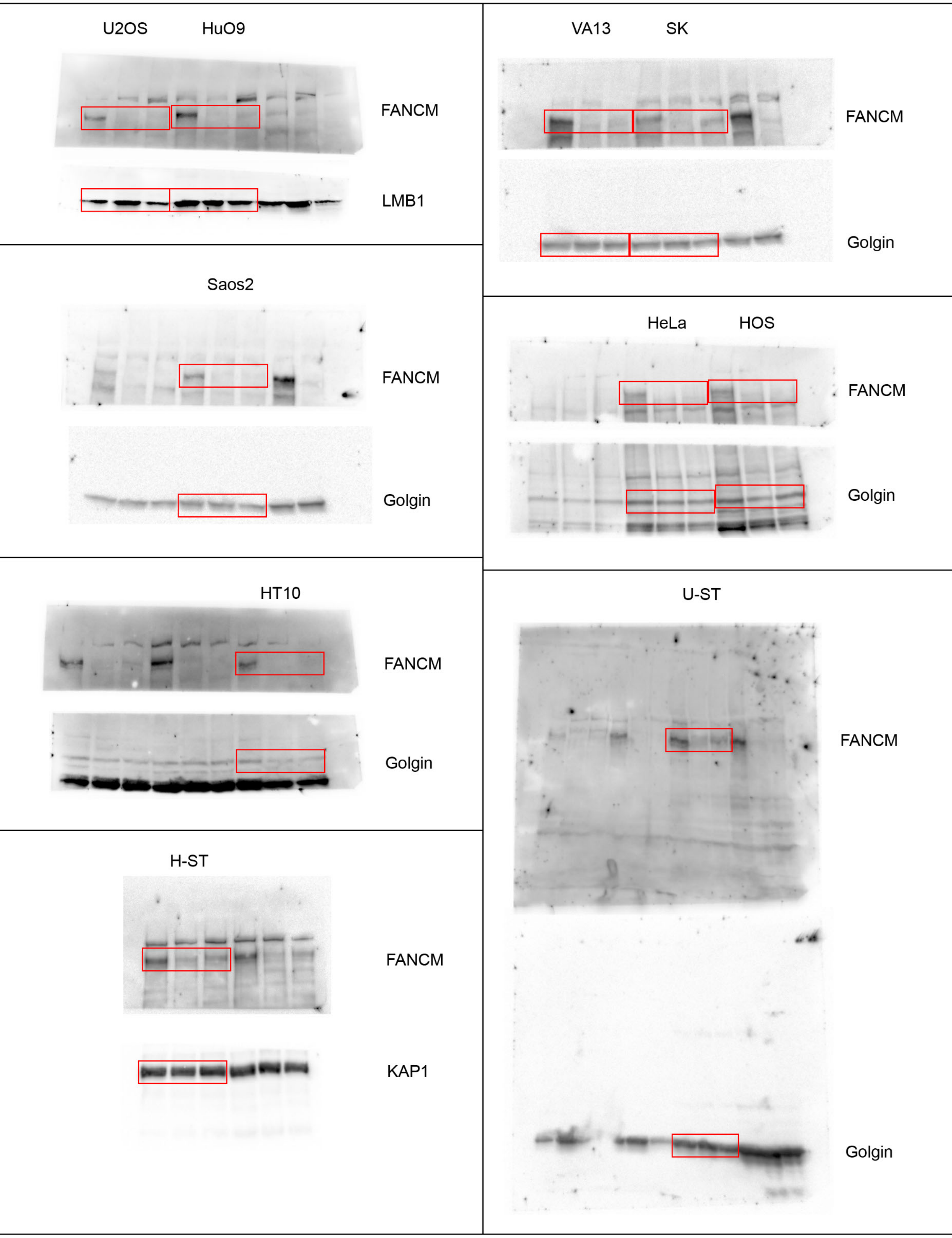

Uncropped blots (related to Fig. 1g and Fig. 2c)

Fig. 1g

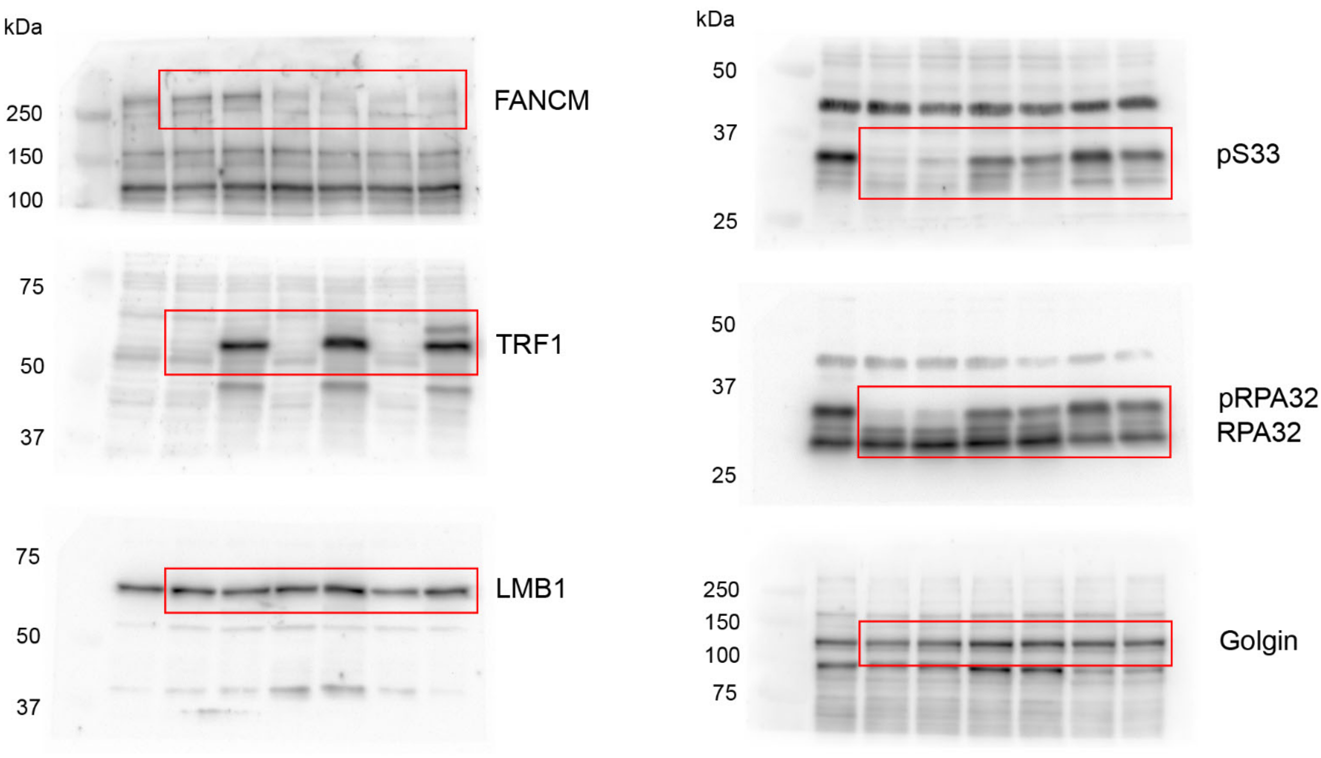

Fig. 2c

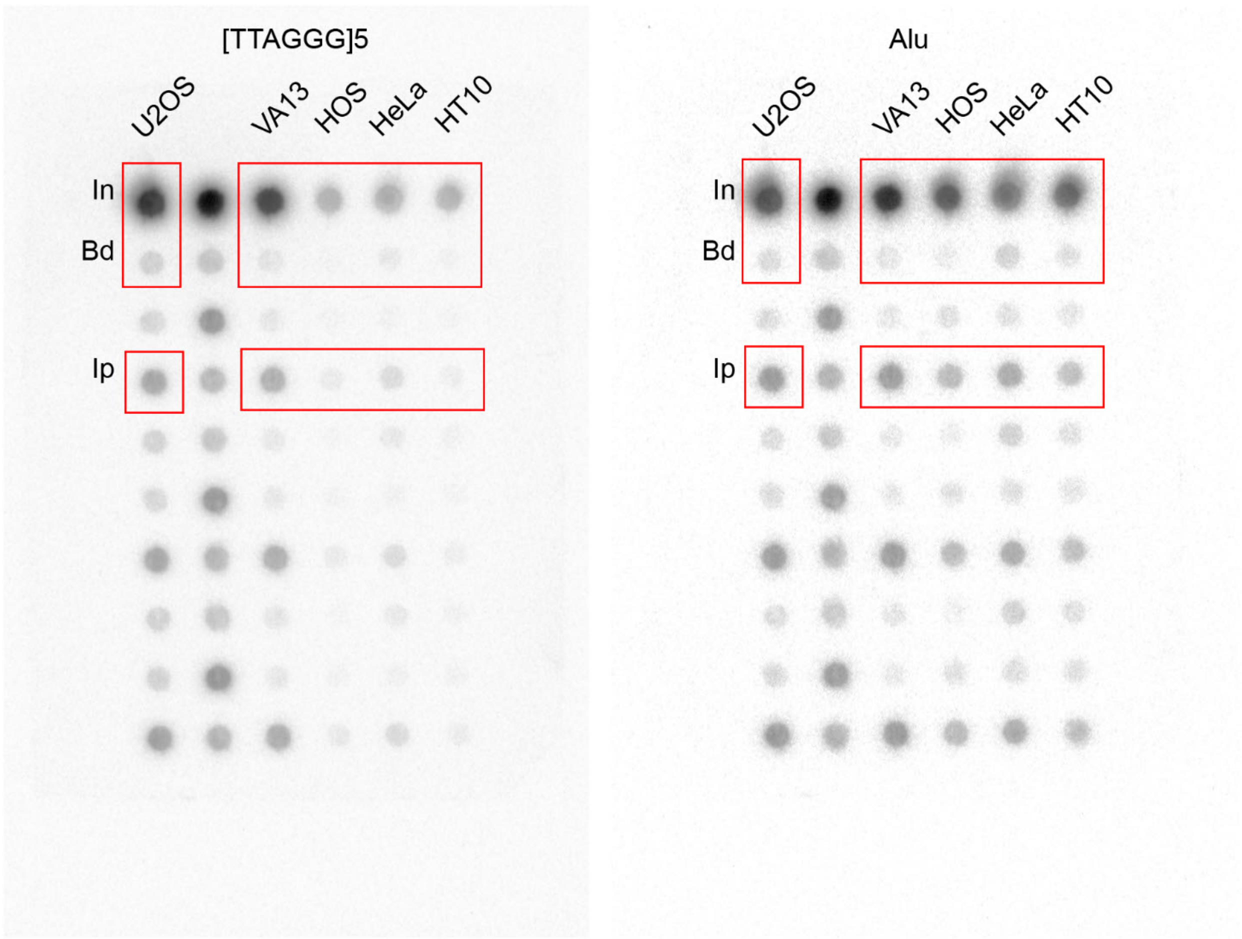

Uncropped blots (related to Fig. 2e)

Fig. 2e

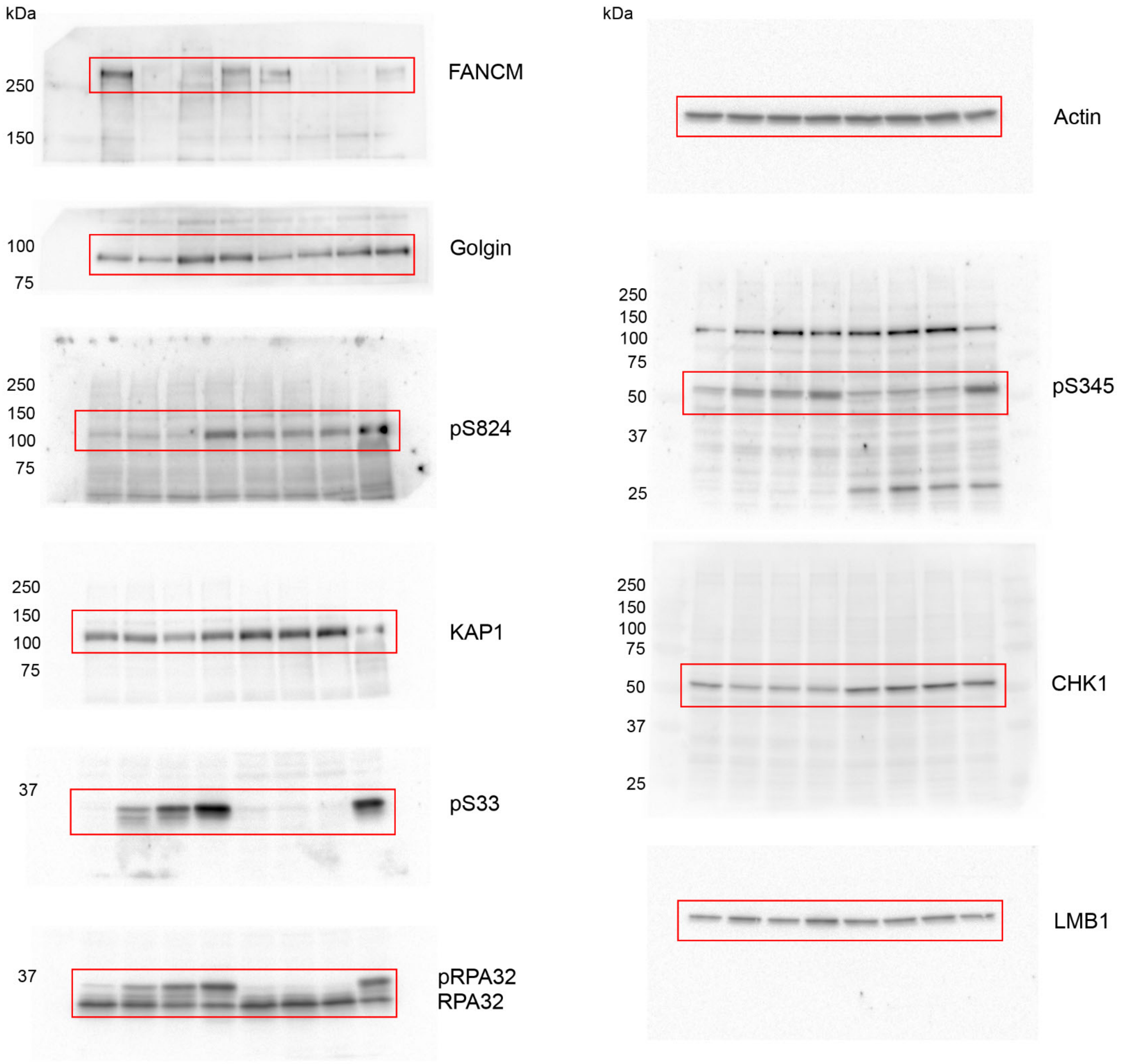

Uncropped blots (related to Fig. 3a)

Fig. 3a

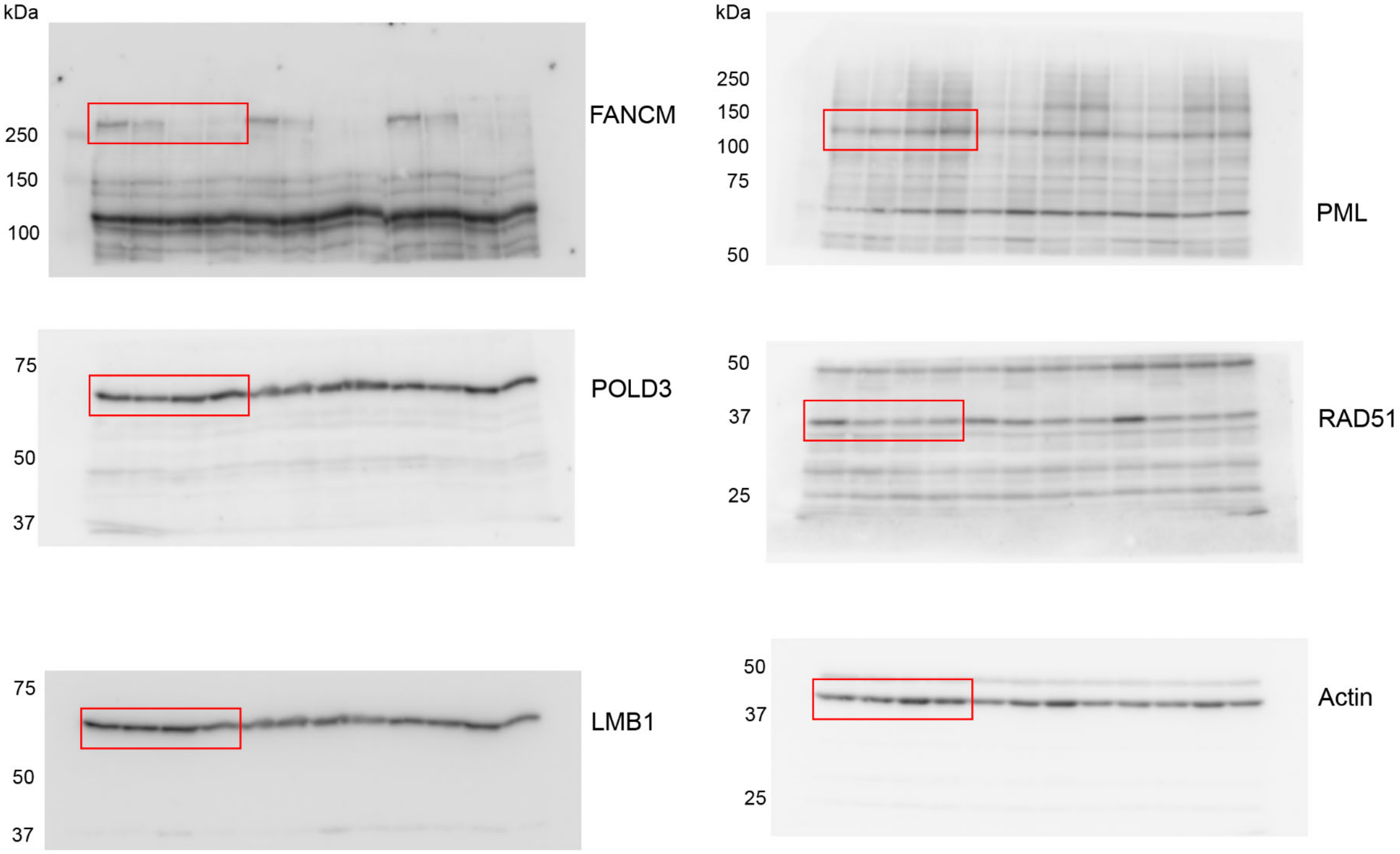

Uncropped blots (related to Fig. 5a)

Fig. 5a

denat Telo 2

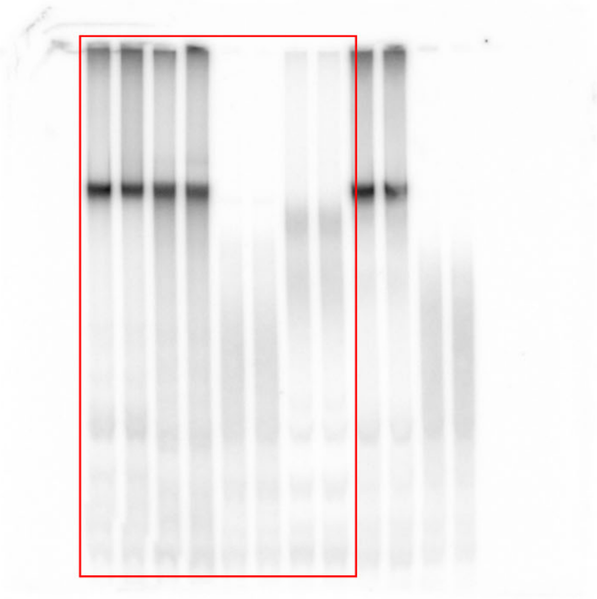

denat Telo 2

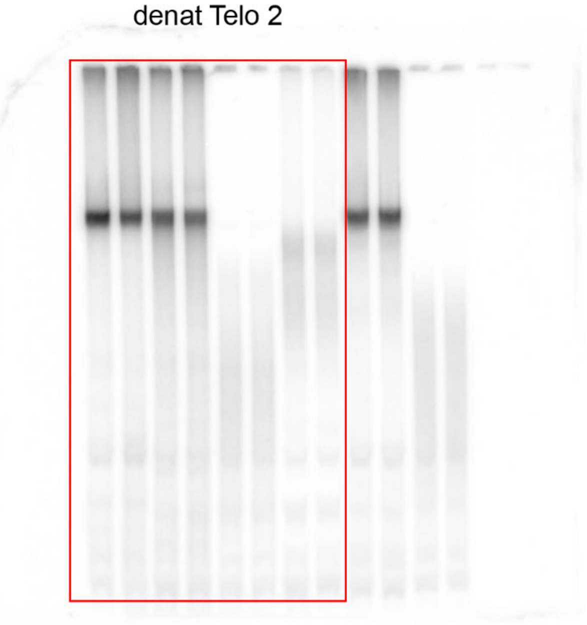

native [TTAGGG]5

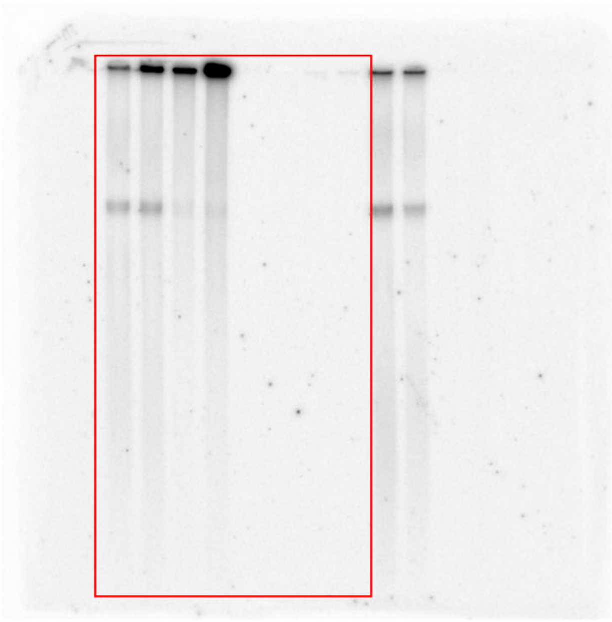

native [CCCTAA]5

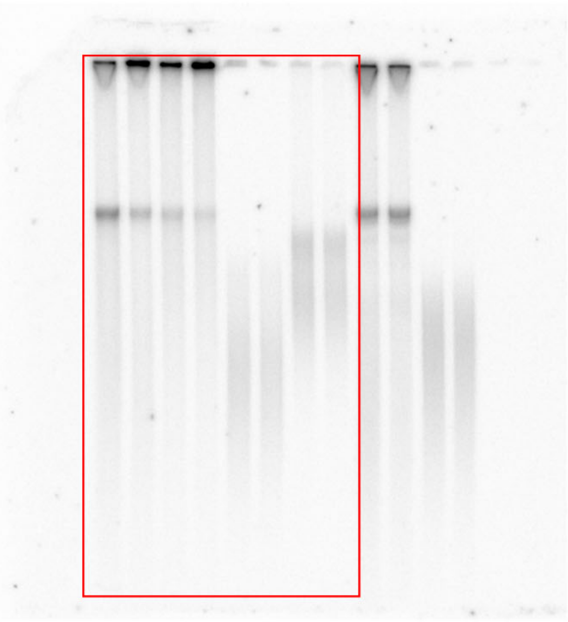

Uncropped blots (related to Fig. 5b)

Fig. 5b

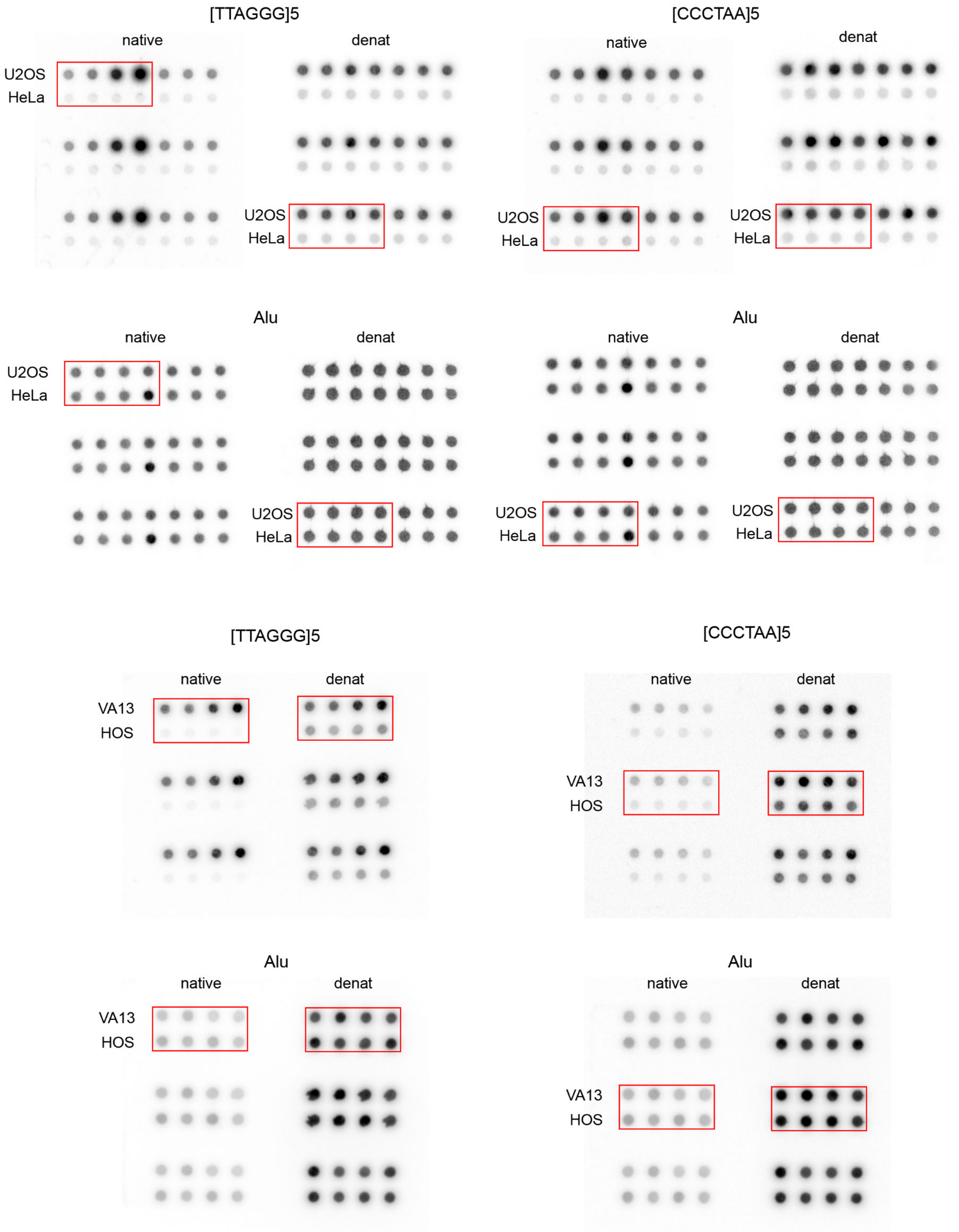

Uncropped blots (related to Fig. 5c and 5d)

Fig. 5c

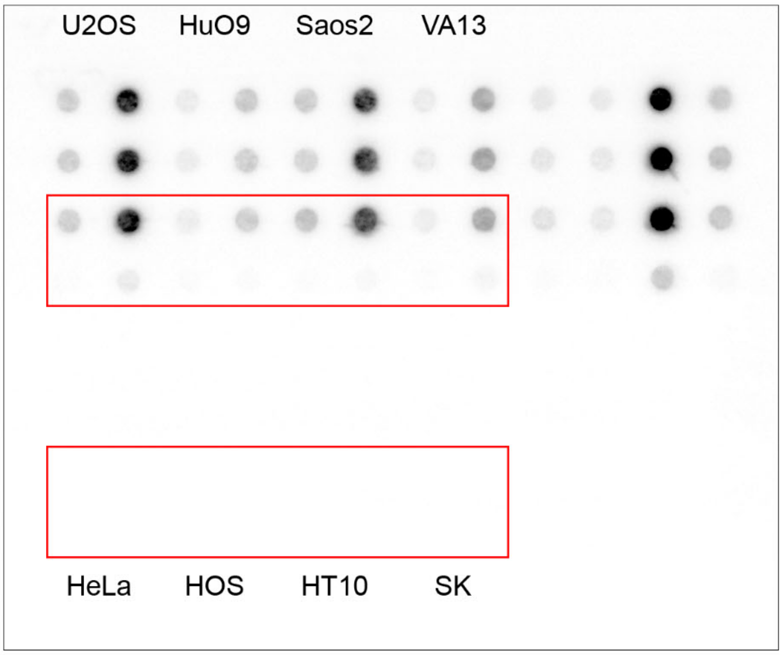

Fig. 5d

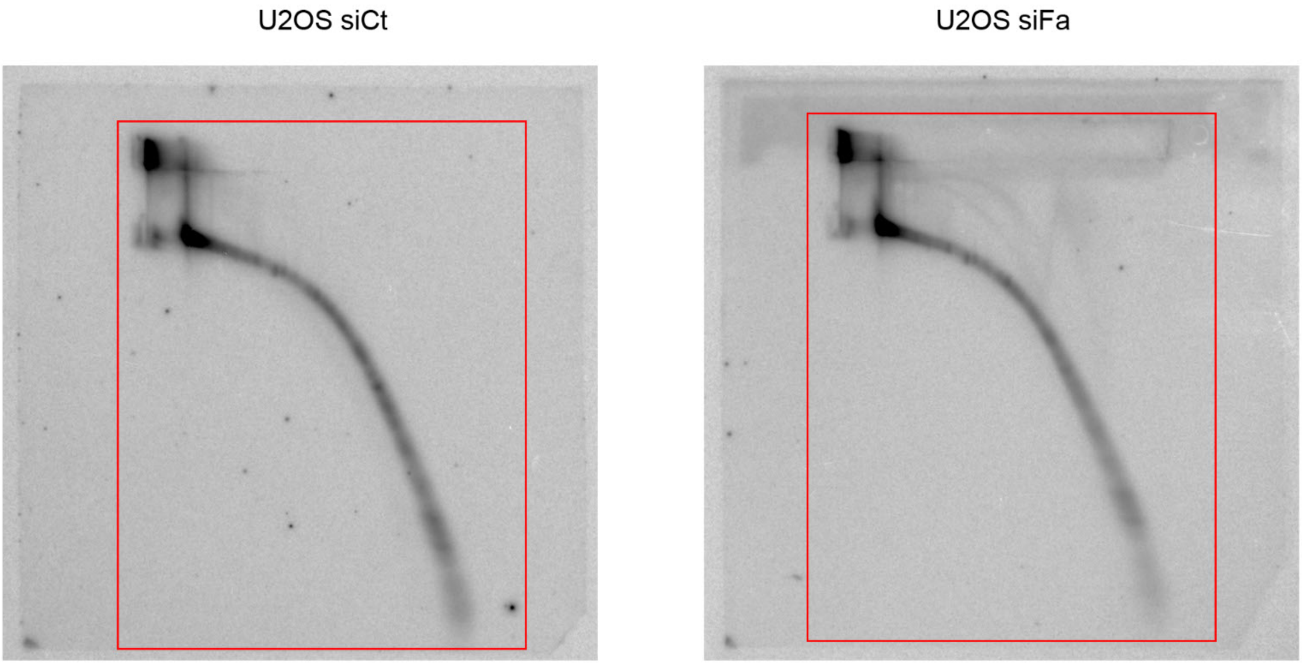

Uncropped blots (related to Fig. 6a and Fig. 7)

Fig. 6a

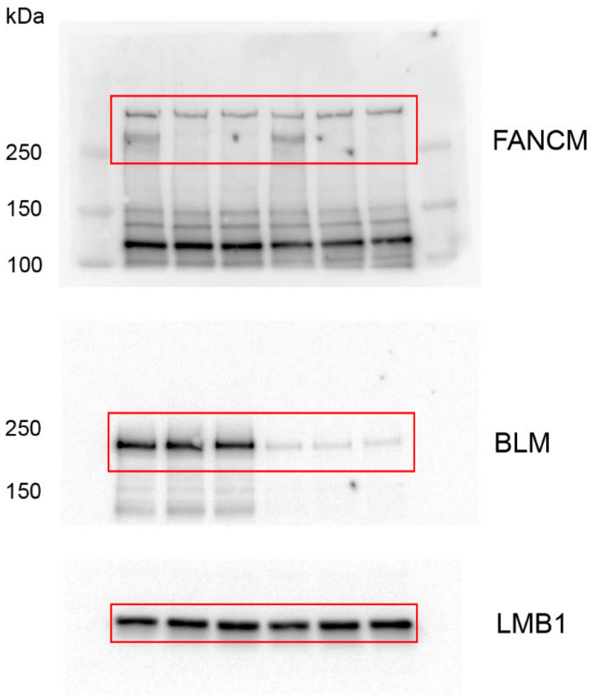

Fig. 7a

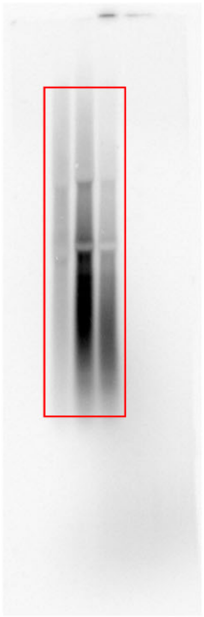

Fig. 7b

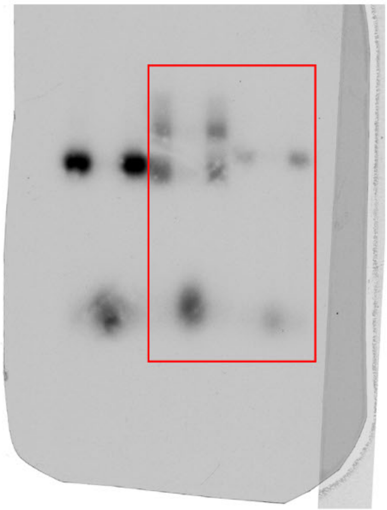

Fig. 7c

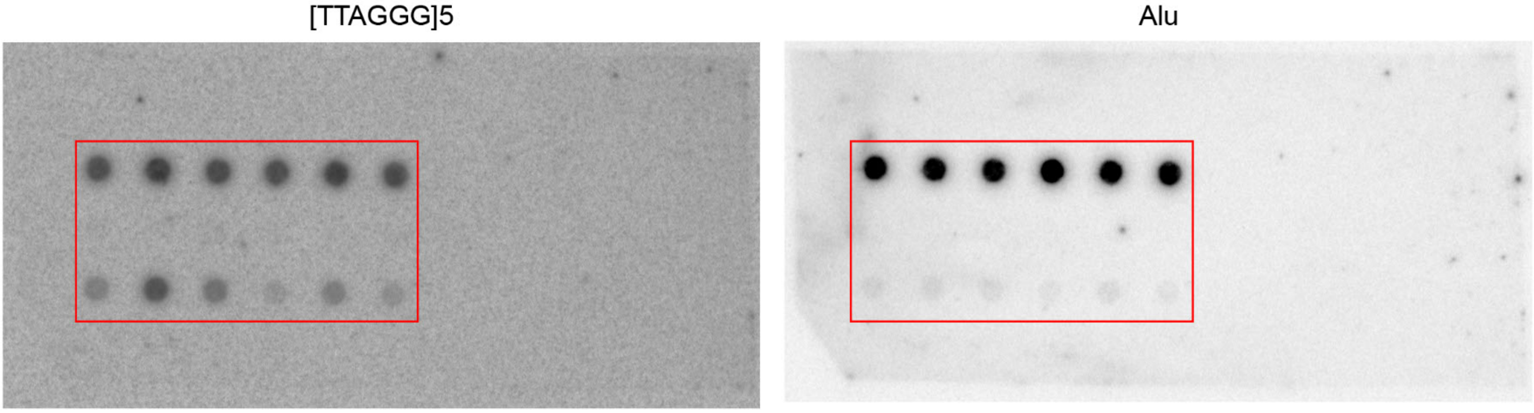

Uncropped blots (related to Fig. 8 and Supplementary Fig. 2b)

Fig. 8a

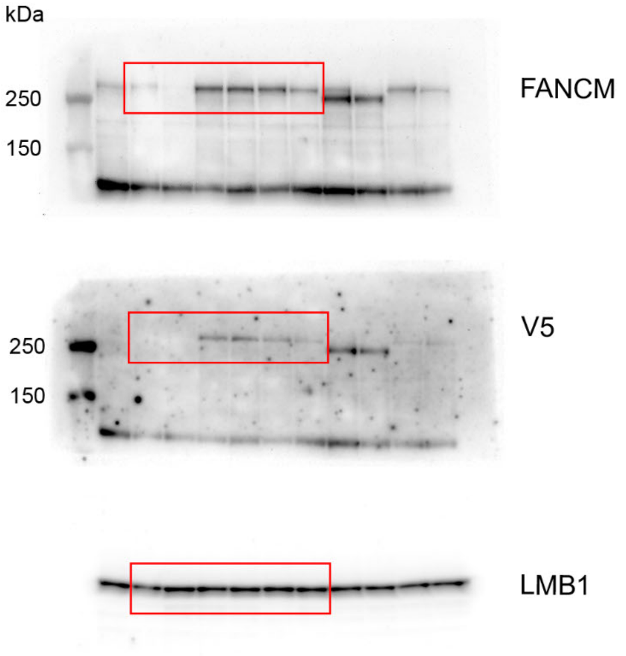

Fig. 8d

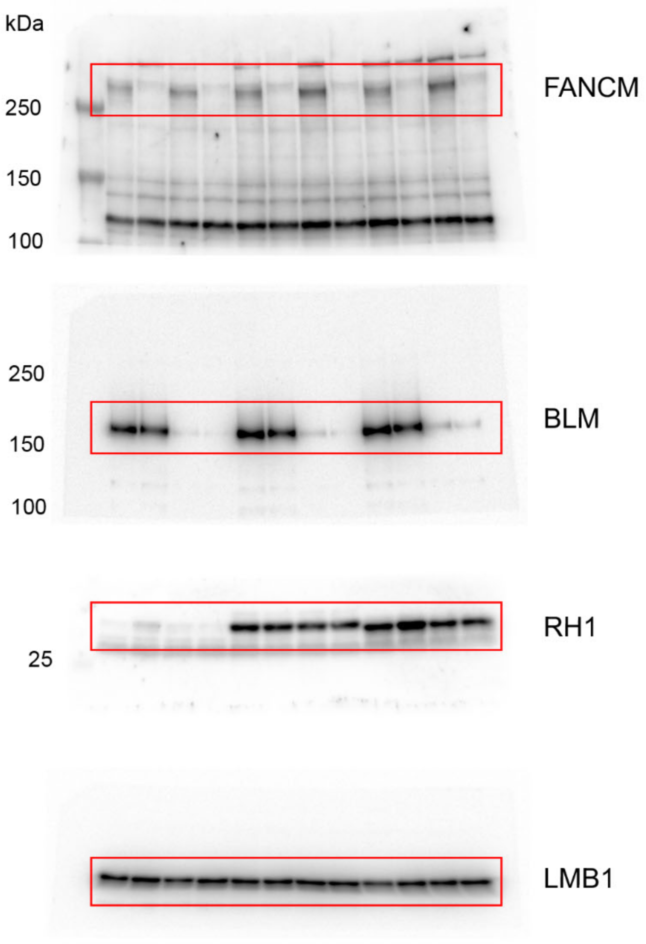

Supplementary Fig. 2b

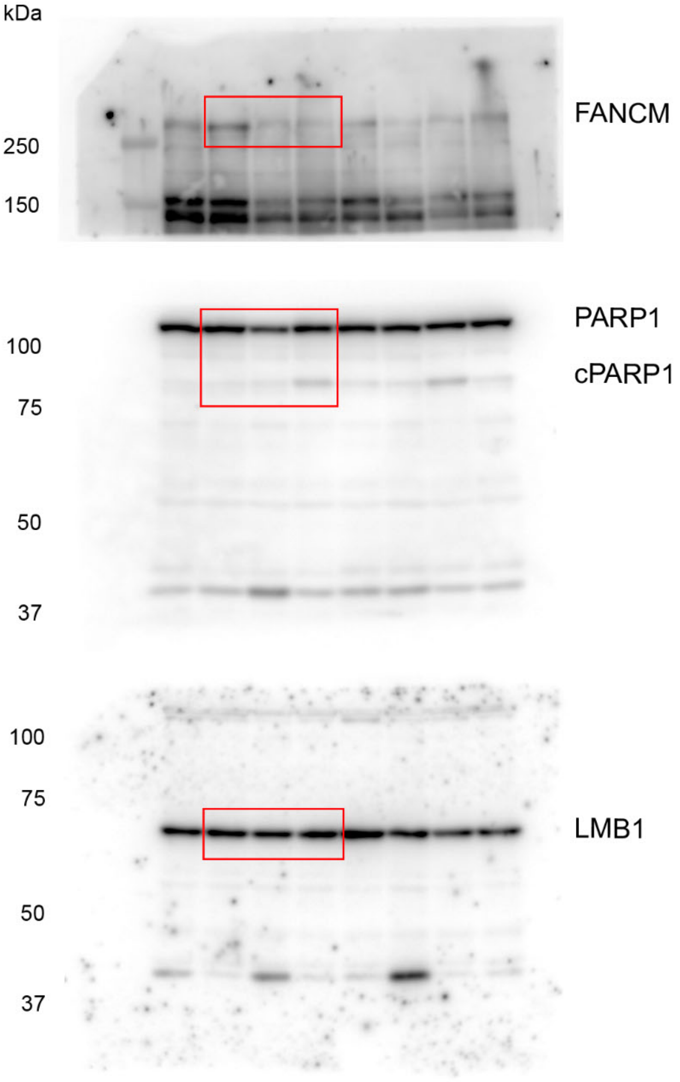

Uncropped blots (related to Supplementary Fig. 4a and 4b)

Supplementary Fig. 4a

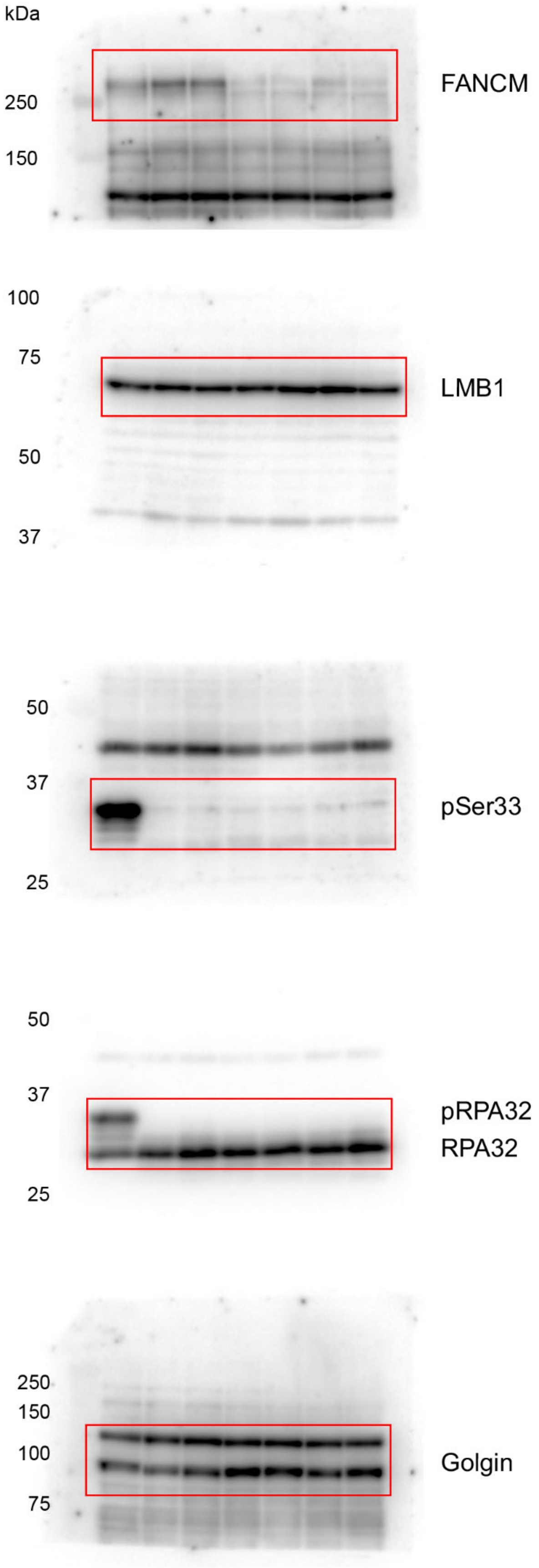

Supplementary Fig. 4b

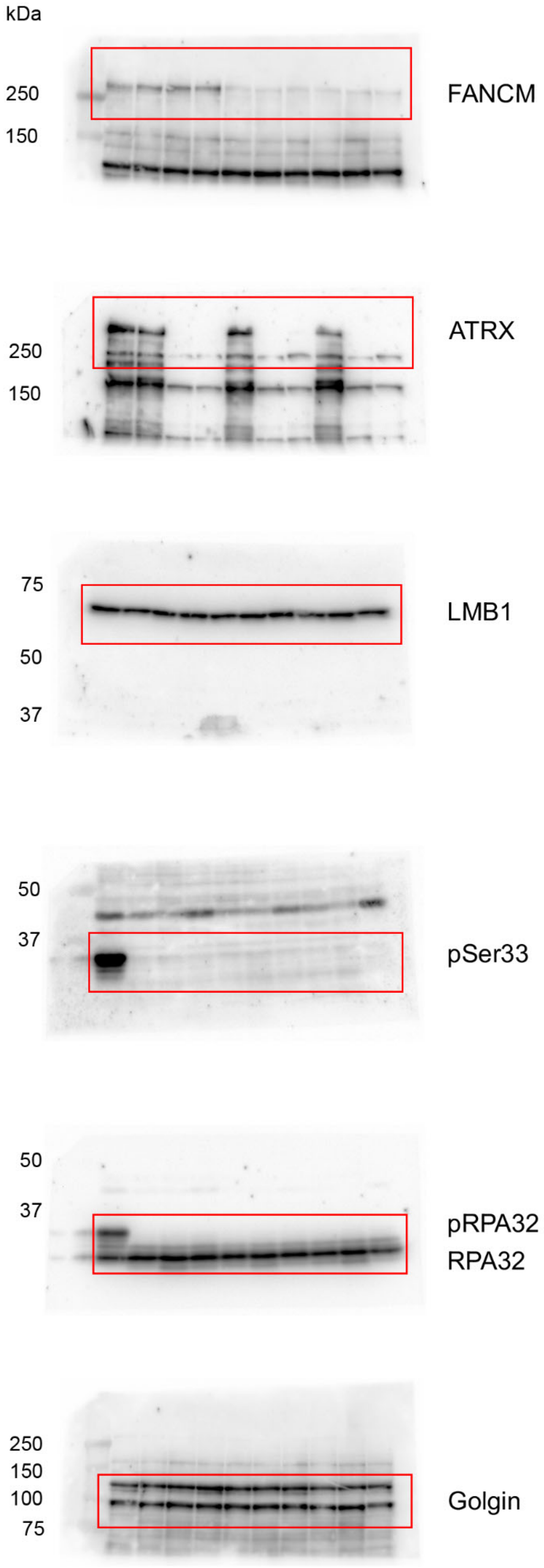

Uncropped blots (related to Supplementary Fig. 4c and 4d)

Supplementary Fig. 4c

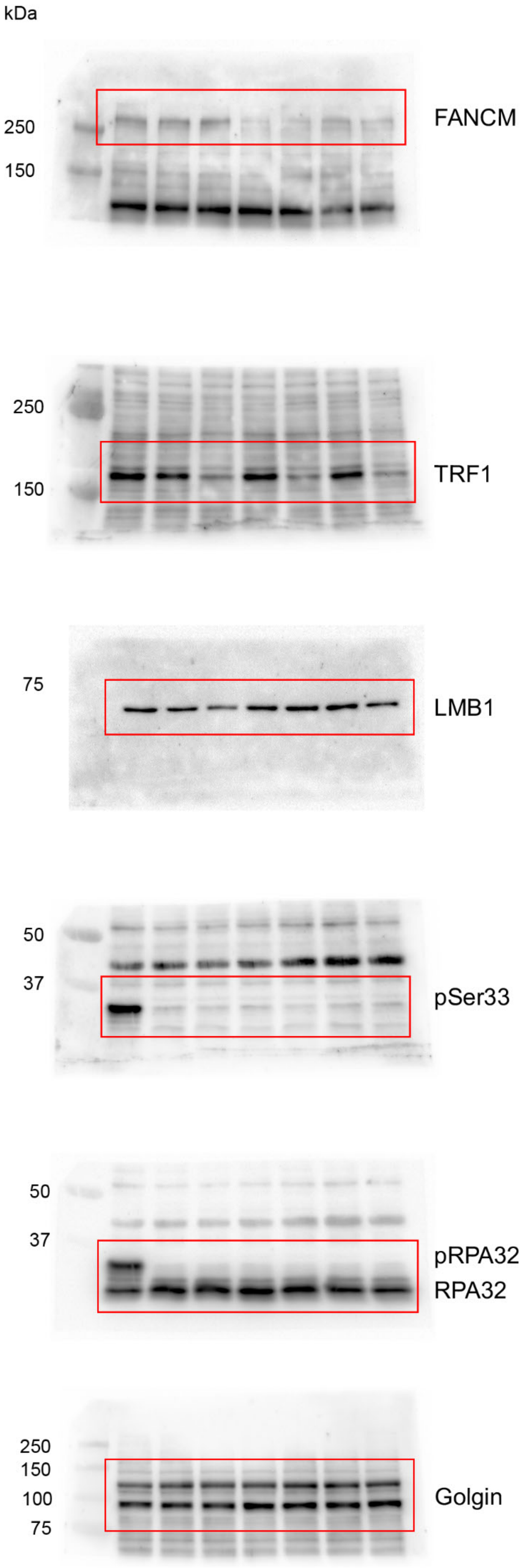

Supplementary Fig. 4d

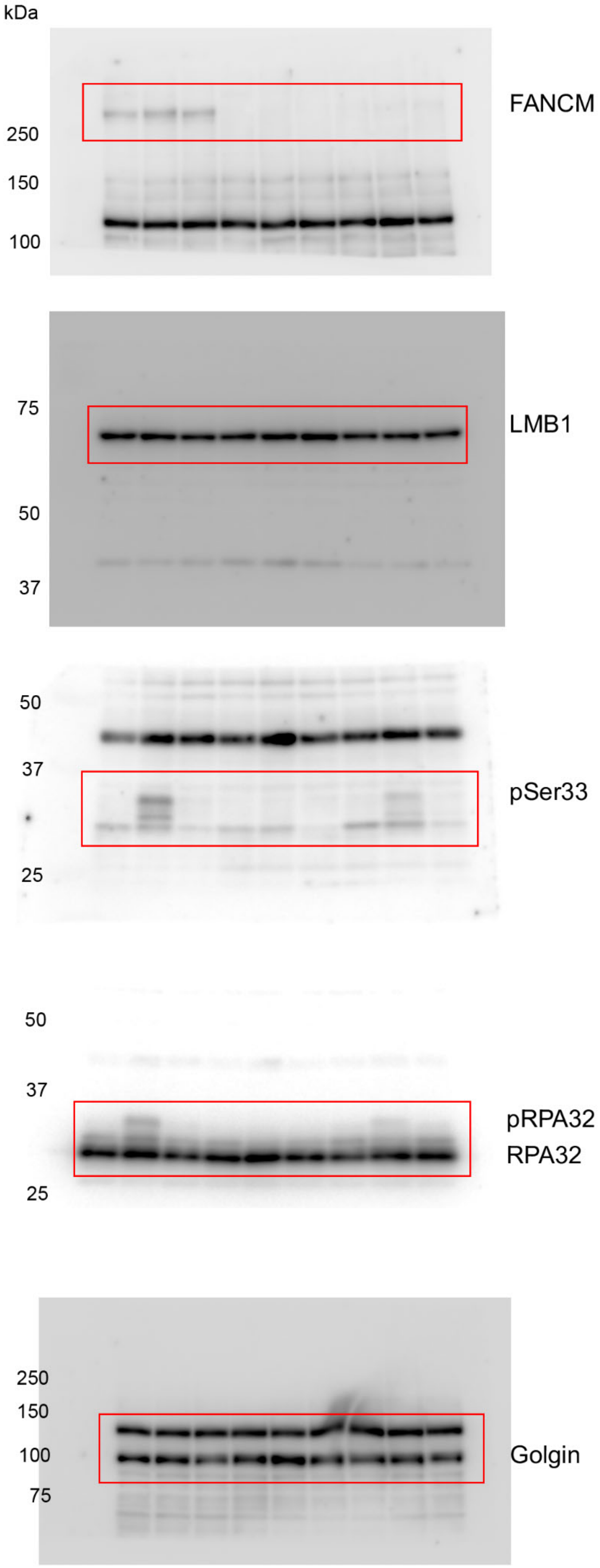

Uncropped blots (related to Supplementary Fig. 6)

Supplementary Fig. 6a

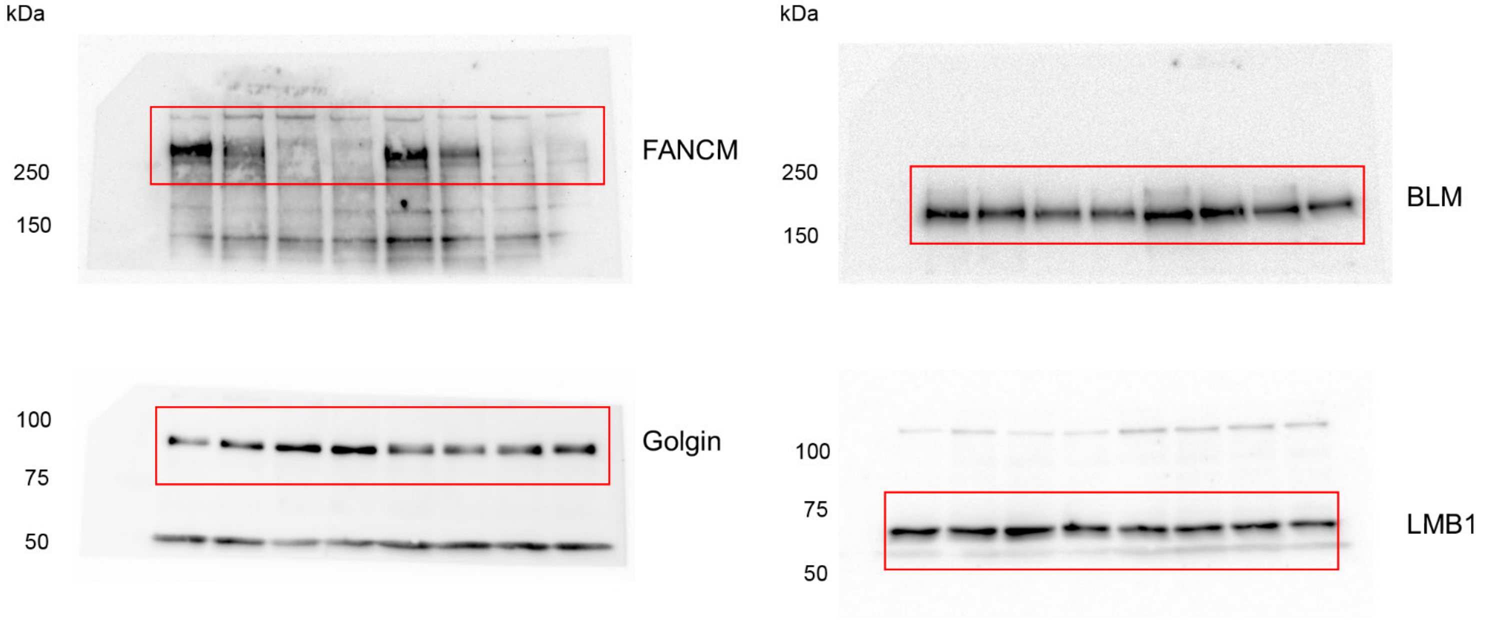

Supplement: Supplementary file 4 — Source Data [file 41467_2019_10179_MOESM4_ESM.zip › Source Data - Uncropped blots.pdf]
